# Lower Urinary Tract Dysfunction in Pediatric Patients with Multiple Sclerosis: Diagnostic and Management Concerns

**DOI:** 10.3390/children11050601

**Published:** 2024-05-16

**Authors:** Maria Laura Sollini, Chiara Pellegrino, Giulia Barone, Maria Luisa Capitanucci, Antonio Maria Zaccara, Leonardo Crescentini, Enrico Castelli, Gessica Della Bella, Federico Scorletti, Laura Papetti, Gabriele Monte, Michela Ada Noris Ferilli, Massimiliano Valeriani, Giovanni Mosiello

**Affiliations:** 1 Division of Neuro-Urology, Bambino Gesù Children’s Hospital, IRCCS, 00165 Rome, Italy; marialaura.sollini@alumni.uniroma2.eu (M.L.S.); giulia.barone@opbg.net (G.B.); mluisa.capitanucci@opbg.net (M.L.C.); antoniomaria.zaccara@opbg.net (A.M.Z.); leonardo.crescentini@opbg.net (L.C.); giovanni.mosiello@opbg.net (G.M.); 2 Clinical Science and Translational Medicine, Tissue Engineering and Remodeling Biotechnologies for Body Function PhD School, University of Rome Tor Vergata, Via Cracovia 50, 00133 Rome, Italy; 3 Pediatric Surgery, University of Genoa, DINOGMI, Largo Paolo Daneo 3, 16132 Genoa, Italy; 4 Neurorehabilitation Unit, Bambino Gesù Children’s Hospital, IRCCS, 00165 Rome, Italy; enrico.castelli@opbg.net; 5 Neurorehabilitation and Adapted Physical Activity Day Hospital, Bambino Gesù Children’s Hospital, IRCCS, 00165 Rome, Italy; gessica.dellabella@opbg.net; 6 Neonatal Surgery Unit, Bambino Gesù Children’s Hospital, IRCCS, 00165 Rome, Italy; federico.scorletti@opbg.net; 7 Developmental Neurology Unit, Bambino Gesù Children’s Hospital, IRCCS, 00165 Rome, Italy; laura.papetti@opbg.net (L.P.); gabriele.monte@opbg.net (G.M.); michela.ferilli@opbg.net (M.A.N.F.); massimiliano.valeriani@opbg.net (M.V.)

**Keywords:** multiple sclerosis, pediatric, neurogenic bladder, LUTS, LUTD, neurogenic bowel

## Abstract

Background: Multiple sclerosis (MS) is increasing in the pediatric population and, as in adults, symptoms vary among patients. In children the first manifestations can sometimes overlap with acute neurological symptoms. Urological symptoms have not been much studied in childhood. We shared our experience with MS urological manifestation in children. Methods: This article is a retrospective evaluation of all children with MS, according to the Krupp criteria, who also present with urological symptoms. We collected demographic and clinical history, the MR localization of demyelinating lesions, urological symptoms, and exams. Results: We report on six MS pediatric cases with urological manifestation. Urinary symptoms, characterized by urinary incontinence in five patients and urinary retention in one patient, appeared in a different time frame from MS diagnosis. Urodynamic exams showed both overactive and underactive bladder patterns. Treatment was defined according to lower urinary tract dysfunction, using clean intermittent catheterization, oxybutynin, and intradetrusor Onabotulinum Toxin-A injection. A low acceptance rate of invasive evaluation and urological management was observed. Conclusions: The MS diagnosis was traumatic for all our patients. We believe it is important to address urological care in young people from the time of diagnosis for prompt management; it could be useful to include a pediatric urologist in multidisciplinary teams.

## 1. Introduction

Multiple sclerosis (MS) is a chronic, inflammatory, autoimmune condition characterized by the gradual degeneration and loss of myelin within the central nervous system, resulting in demyelination; it is a leading cause of permanent disability in young adults, second only to trauma [1].

More than 2.8 million people in the world are affected by MS, and Italy is considered a high-prevalence country, with over 200 cases per 100,000 [2]. Although MS is typically diagnosed in young adults, its prevalence in pediatrics is increasing globally. Forms of MS with onset in pediatric patients (<16-year-olds or sometimes <18-year-olds) are defined as POMS (Pediatric Onset Multiple Sclerosis) or EOMS (Early Onset MS) or juvenile MS [3,4,5]. Pediatric multiple sclerosis occurs at a rate of 0.13 to 0.66 cases per 100,000 children annually, with up to 10% of multiple sclerosis cases experiencing their initial demyelinating episode before reaching 18 years of age; higher prevalence is recorded in children of 13 to 16 years and in female patients >10 years old, while before 10 years of age, the number of MS cases are similar in boys and girls. Later, the preponderance of female patients is 4.5:1, maybe because of the start of menstruation and hormonal changes [3,4,6]. MS is a rare diagnosis at a prepubertal age, comprising less than 1% of all cases [4,6].

In POMS, clinical manifestations can be very varied between patients and can have a different clinical course to that of adult MS; 98% of pediatric MS patients exhibit a relapsing–remitting (RR) course to a greater extent than is observed in adult-onset MS cases [3]. Typically, patients present with neurological symptoms such as headaches, nausea, motor and sensitive hemisyndrome, and cerebellar disfunction following a viral infection [7], which can lead physicians to mistakenly diagnose an acute central nervous system disorder, instead of MS. The International Pediatric Multiple Sclerosis Study Group (IPMSSG) published in 2007 the first diagnostic criteria for POMS, and these were revised in 2012. These criteria referred to the 2010 McDonald criteria for the demonstration of dissemination in time and space [8,9].

MS can also cause lower urinary tract dysfunction (LUTD), present in 10% of patients at the time of MS diagnosis and in 80% after 10 years of disease, with a possible strong impact on the quality of life, as well as on the upper urinary tract and therefore on renal function [10,11]. Poor levels of data are present in the literature regarding urological clinical patterns in pediatric MS patients. Most works about pediatric MS do not investigate bladder and bowel function; therefore, the incidence rate of these symptoms is extremely variable (from 1 to 39%) [5,6].

The aim of our work is to present our experience on the diagnosis and management of urological manifestations in children with MS.

## 2. Materials and Methods

We retrospectively reviewed the clinical charts of all patients with MS diagnosis and urological symptoms evaluated at our pediatric urology division in the last five years, from January 2019 to January 2024. We included all patients with MS and urological symptom onset before the age of 18 years. In accordance with our institutional guidelines, written consent was obtained from parents.

MS was diagnosed according to the Krupp criteria [12], shown in Table 1 and Table 2.

We collected demographic and clinical data, including onset symptoms, the magnetic resonance (MR) localization of demyelinating lesions, and the timing of onset and type of urological symptoms. We reviewed all data related to diagnostic pathway and clinical management to critically assess our approach.

## 3. Results

Among the 81 patients with a diagnosis of MS carried out at our hospital, 6 patients presented urinary symptoms and were referred to our division of pediatric urology. The incidence of LUTD in our series was 7.4%.

Of these patients, four were female and two were male. The median age at time of MS diagnosis was 14.55 years (range of 11.4 to 17.3 years). Urinary symptoms were already present at the time of MS diagnosis in two patients (one with a worsening of known voiding problems during MS onset); in three patients, the symptoms appeared between 6 months to 5 years after MS diagnosis.

Urinary symptoms were characterized by urinary incontinence in five patients (one also had enuresis) and urinary retention in one patient. Urinary ultrasounds showed the absence of urinary tract dilatation in all patients.

Three patients underwent invasive urodynamic exams that diagnosed an overactive bladder (OAB) in one case, acontractile detrusor in another, and detrusor–sphincter dyssynergia in the last case. Two patients showing indirect signs of dyssynergia with pathological post-voiding residual underwent non-invasive urodynamic evaluations only, due to lack of patient collaboration; the male patient presented symptom resolution following the remission of an acute attack of MS, so no invasive urodynamic exam was performed. One patient, recently referred to our division, is scheduled for invasive urodynamic.

Regarding the management of LUTD, one patient refused any urological therapy and was lost at follow-up. The remaining five patients were initially managed with standard urotherapy; in one of these cases, the urological manifestations were resolved completely due to the change in MS therapy and standard urotherapy. Standard urotherapy involves various elements, including behavioral instructions (fluid intake, diet, regular voiding, bowel habits) and clarification about urological anatomy and functioning. Three patients needed clean intermittent catheterization (CIC), of which two were also undergoing pharmacological therapy with oxybutynin and intradetrusor Onabotulinum Toxin A (BTX-A) injections; of these patients, one girl also underwent pelvic floor biofeedback therapy, without success.

The localization of demyelinating lesions does not seem to be correlated with the onset of urological manifestations.

Three patients reported constipation, and one of these was the boy with previous myelomeningocele. Two of these were successfully managed with TAI (trans-anal irrigation); one declined therapy. No patients reported fecal incontinence.

Patient characteristics and follow-ups are summarized in Table 3.

### 3.1. Case Serie

#### 3.1.1. Patient 1

A 13.10-year-old girl was first admitted to our hospital presenting with persistent and progressive walking and balance disorders.

After a thorough anamnesis some details emerged: motor impairments had always been present with progressive worsening over the last year. She had urgency, enuresis, and sometimes urinary incontinence which progressively worsened to the point of needing to wear diapers; also learning difficulties emerged. No family history of neurological pathology was recorded.

Brain and spine MR revealed multiple demyelinating lesions, localized in the subcortical and deep white matter of both cerebral hemispheres, the corpus callosum, the nucleus thalamus–capsular regions, the brainstem, the cerebellum, and in the cervico-dorsal spinal cord. Further diagnostic tests led to MS diagnosis, according to the Krupp criteria [12].

Due to the urological symptoms found during the neurological examination, she was referred to our urological division, but the patient only agreed to start a urological diagnostic path one year later. No urinary tract infections were mentioned in her history.

Urinary US showed a thickened bladder wall without upper urinary tract (UUT) dilatation. The urodynamic exam diagnosed an OAB with small bladder capacity and high detrusor pressures; treatment with oxybutynin and CIC was started. Initially, there was poor patient compliance with CIC, with progressively greater adherence over time, regularizing the frequency of catheterization during the day. A pelvic floor biofeedback cycle was also performed, without any clinical or urodynamic benefit.

At 18 months of evaluation, despite CIC, oxybutynin, and pelvic floor muscle training therapy, urinary incontinence persisted. An intradetrusor Onabotulinum Toxin A (BTX-A) endoscopic injection was carried out. During cystoscopy, bladder walls were hyperemic, without trabeculae; the histological examination of the bladder wall biopsy revealed moderate chronic cystitis.

At last follow-up, an improvement in the symptoms was recorded, without any urinary tract infections; urinary US showed normal UUT with persistent thickened bladder walls and renal function was normal. She is now scheduled for a second BTX-A treatment.

During the examination, the presence of severe constipation, without fecal incontinence, emerged, for which the patient has been prescribed the use of TAI, which never performed because of patient refusal.

#### 3.1.2. Patient 2

A 15.6-year-old girl, previously treated due to severe familial obesity, was admitted to our hospital because of the recent onset of walking disorders and left leg paresthesia.

With a thorough anamnesis emerged a history of 2 years of balance alterations and a worsening of the known obesity due to the inability to practice sports because of movement disorders. The patient was being treated by a psychologist for anxiety disorder.

Brain and spine MR revealed multiple demyelinating lesions, localized in the bilateral deep periventricular white matter, subcortical white matter, corpus callosum, bulb, pons, upper and middle cerebellar peduncles, and in the cervico-dorsal spinal cord. Further diagnostic tests led to MS diagnosis, according to the Krupp criteria [12].

Six months after diagnosis, the girl began to experience micturition symptoms, including one episode of acute urinary retention that required catheterization and urinary tract infections. Further urological examination was recommended by neurologist.

An abdominal US revealed no dilatation of the upper urinary tract but significant post-void residual. In terms of voiding, cystourethrography intermittent voiding with high post-void residual and an absence of vesicoureteral reflux or urethrography alteration were found. The urodynamic exam led to a diagnosis of a neurogenic bladder with dyssynergy and significant post-void residual. CIC was prescribed; initially, it was performed discontinuously but was subsequently accepted by the patient. Upon the regularization of the CIC, a resolution of the urinary symptoms was noted.

Constipation, present since childhood, worsened following MS diagnosis and required treatment with osmotically acting laxatives and TAI.

#### 3.1.3. Patient 3

A 17.3-year-old boy with spina bifida received a MS diagnosis after the worsening of the known neurological and urological symptoms (neurogenic bladder with incontinence).

This patient had been treated at our hospital from early childhood in a multidisciplinary setting due to myelomeningocele (surgically removed at birth at another hospital), anorectal malformation (initially managed at another hospital and then referred to our institute), secondary tethered cord (discovered due to the appearance of walking impairment and subjected to a detethering surgical procedure at 12 years of age), and a left neurogenic cavus foot (surgically treated).

Due to his known neurogenic bladder with acontractile detrusor and low bladder capacity, discovered through invasive urodynamic evaluation, the patient had been managed with CIC since early childhood; subsequently, pharmacological therapy (with oxybutynin), then endoscopic injections of bulking agents in the bladder neck and intratradetrusor therapy with BTX-A were added. An attempt at positioning a sacral neuromodulator failed due to a lack of clinical response (urinary and fecal) to the pre-implantation test.

Because of the reduction in sensitivity in the lower limbs and a worsening of urinary incontinence, the patient underwent a new spinal MRI to evaluate any new spinal tethering; MS was instead diagnosed, according to the Krupp criteria [12].

After being lost to urological follow-up for a few years, he was taken back after the diagnosis of MS. He exhibited a progressive decrease in CIC number/daily; the performance of spontaneous micturition with abdominal pressure; the persistence of urinary incontinence; and no urinary tract infections. Renal function was normal, and no alterations were found at urinary tract US. The video-urodynamic test was then repeated, confirming the diagnosis of a neurogenic bladder with acontractile detrusor, reduced bladder capacity, and regular compliance; no vesicoureteral reflux was recorded. The BTX-A treatment was then resumed with a good but partial clinical response, and the patient is waiting to undergo a new therapeutic program.

Following the diagnosis of MS, constipation also worsened, needing an increase in pharmacological therapy with osmotically acting laxatives and TAI.

#### 3.1.4. Patient 4

A 13.10-year-old girl was initially admitted to our hospital because of frequent episodes of fatigue, headaches (unilateral, frontal, moderate-severe) with phonophobia and nausea, and upper limb paresthesia. A brain and spine MR showed multiple demyelinating lesions, localized in the anterior frontal and left mesial temporal subcortical region, semioval centers, paraventricular region, corpus callosum splenium, bulb, and cervico-dorsal spinal cord. Further diagnostic tests led to MS diagnosis, according to the Krupp criteria [12].

She also had a positive genetic evaluation for celiac disease and difficulty with attention and anxiety.

Approximately three years after the initial diagnosis, she developed urinary symptoms characterized by daily urine leakage. The non-invasive urodynamic test showed an interrupted flow with a low voided volume according to age and significant post-voiding residual, for which oxybutynin therapy and percutaneous tibial nerve stimulations (PTNS) were prescribed. The proposed therapy was not followed due to patient refusal, and she was lost at follow-up.

She did not report any issues with constipation or fecal incontinence.

#### 3.1.5. Patient 5

A 16.8-year-old girl was initially admitted to a local hospital for a recent onset of lack of concentration, imbalance, and walking disorders with recurrent falls. A brain and spine MR showed multiple demyelinating lesions, localized in the periventricular white substance, semioval centers, pons and midbrain, left middle cerebellar peduncle, and the cervico-dorsal spinal cord. She was then referred to our hospital for further diagnostic tests which confirmed an MS diagnosis, according to the Krupp criteria [12].

She was recently referred to our division due to urinary leakage, which began 1 year after MS diagnosis. She is currently scheduled for a urodynamic exam.

No constipation or fecal incontinence were reported.

#### 3.1.6. Patient 6

An 11.4-year-old boy was initially admitted to a local hospital for the acute onset of diplopia due to the paralysis of the III, IV, and VI cranial nerves, which had regressed after corticosteroid and immunoglobulin administration. A brain and spine MR showed multiple demyelinating lesions, localized in the periventricular regions, bulbs, and the cervical-dorsal spinal cord. After the interruption of corticosteroids, bitemporal headaches, photophobia, and widespread pain appeared. He was then referred to our hospital where, following more tests, the diagnosis of MS was confirmed, according to the Krupp criteria [12].

Five years later, during a flare-up of the disease, urinary symptoms emerged, characterized by urgency and occasional urge incontinence. The abdominal US showed no dilatation of the upper urinary tract or other urinary system anomalies. The non-invasive urodynamic examination showed a mild voiding dysfunction with elevated bladder capacity and a post-voiding residual of 10%; he was discharged with timed voiding instructions. One month after MS therapy change and the remission of the acute MS phase, the resolution of the urological symptoms was also recorded. The patient is currently under conservative urological follow-up.

No constipation or fecal incontinence were reported.

## 4. Discussion

In the pediatric population, MS generally appears with focal deficits such as paresthesia and unilateral weakness or numbness but also visual loss, ataxia, and transverse myelitis. Multiple symptoms can be present at disease onset in about 10–67% of cases; optic neuritis can be identified in 0–50% of POEMs, with 10% of patients complaining of visual changes, and polyfocal deficits, encephalopathy, and brainstem involvement are more frequent in patients <10 years old [5,6,14].

The diagnosis of adult-onset MS is based on the revised McDonald criteria. As already mentioned, for pediatric-onset MS, the diagnostic criteria were published by IPMSSG and referred to the 2010 McDonald criteria [12,13]. The McDonald criteria emphasize the exclusion of syndromes that mimic multiple sclerosis and aim to enable earlier diagnosis [15].

An important differential diagnosis, which takes advantage of these diagnostic criteria, is acute disseminated encephalomyelitis (ADEM). ADEM is characterized by focal symptoms too, but it is more common in children than MS [3]. Differentiating between ADEM and MS poses significant challenges. During the years, the number of acute attacks and the time elapsed between episodes of relapses were considered for differential diagnoses [12,16]. Clinical history can be helpful for differentiation: ADEM typically manifests as a monophasic demyelinating disease with seizures or behavioral disorders [4]. It may be induced by viral infection or vaccination, e.g., measles or the Varicella–Zoster virus. When two or more periventricular lesions are observed, coupled with the absence of a diffuse bilateral lesion pattern, the presence of black holes, and the emergence of new lesions in diverse locations during follow-up MRI scans, the likelihood of multiple sclerosis (MS) is significantly heightened [17]. Recent findings indicate that susceptibility-weighted imaging (SWI) could offer utility in distinguishing the initial presentation of pediatric multiple sclerosis from acute disseminated encephalomyelitis (ADEM) [18]. Sometimes, ADEM can acutely present with symptoms such as headache, nausea, vomiting, fever, seizures, an altered state of consciousness, motorsensory hemisyndromes, and cerebellar and brainstem dysfunction that usually lead to the initial diagnosis of meningoencephalitis [7] or other acute nervous manifestations, resulting in a delay in the MS diagnosis.

The most common form of MS is the relapsing–remitting one. In pediatric MS, the relapse rate is described as higher than in adult patients; 60% relapse during the first year. On the other hand, pediatric symptoms during relapsing can be more severe than adult symptoms but they appear more transitory and remit more quickly than in adult-onset MS. In the KIDMUS pediatric MS prospective study, optic neuritis, age >10 years, and multiple lesions (periventricular or subcortical) at MR were defined as predictors of a second attack of MS. An interval of <1 year between two relapses and incomplete recovery after the first episode are negative prognostic factors, signaling an increased risk of reaching a greater disability status [3,5,6,7,14,15]. Because of the relapsing–remitting nature of POMS, after the diagnosis, a repetition of MR is always performed during the follow-up.

An important aspect that differentiates POMS from adult-onset MS is the greater cognitive impairment recorded in the pediatric forms, as highlighted by the 5-year study of Amato et al., which showed a cognitive impairment index deterioration in 56% of patients, an improvement in 25%, and stability in 18.8% [3,19].

One of the least socially accepted manifestations of patients with MS is voiding dysfunction, mainly represented by irritative and obstructive symptoms [11]. Giannantoni et al. found an interesting relationship between presenting symptoms of voiding disfunction and a higher number of urinary disorders and more severe urodynamic alterations [11]. Some authors have highlighted a correlation between the presence and clinical severity of bladder dysfunction and the duration of MS [20]. For this reason, a urological evaluation is recommended concurrently with neurological follow-up, after the repetition of spine MR. After MS diagnosis, we usually perform a follow-up MR after 6 months and then once a year, but the timing of radiological control may vary based on the clinical conditions and the state of activity of the disease.

LUTD is reported in about 50–90% of patients with MS [11]. Irritative symptoms, such as frequency and urge incontinence, are present with a prevalence of 37–99% [10]; these symptoms reflect an OAB pattern at urodynamic exam, which is the most frequent report in MS patients, ranging from 34 to 99% of these UD exams. On a clinical level, another manifestation of OAB can be the recurrence of urinary tract infections, a reduction in which has been demonstrated following the correct management of detrusor overactivity using intradetrusor BTX-A injections [20,21,22,23].

Voiding symptoms include poor streaming, hesitation, and urinary retention; obstructive symptoms are present in 34–79%, potentially resulting in chronic urinary retention. Overflow incontinence can be due to a significant amount of post-void residual. Detrusor underactivity is described in a mean of 25% of MS urodynamic tests (ranging from 0 to 40%), while poor bladder compliance is present in 2–10%. Also, the diagnosis of detrusor–sphincter dyssynergia is widely prevalent, ranging from 3–83%, because of different diagnostic methods. Since MS affects multiple central nervous system regions and it is a progressive disease, a wide spectrum of urodynamic diagnosis has been recorded. It is known that OAB can be caused by brain lesions, detrusor underactivity can be caused by sacral/peripheral lesions, and complex bladder patterns can be caused by partial spinal cord lesions. Irritative and obstructive manifestation is present simultaneously in about 59% male and 51% female patients; in these cases, a combined therapeutic approach is necessary, for example, using CIC for urinary retention symptoms and pharmacological therapy for OAB (e.g., oxybutynin and BTX-A). As a first line approach in the management of all LUTD, urotherapy is fundamental. It includes all non-pharmacological and non-surgical approaches. Standard urotherapy starts with a depth clarification of urological signs and symptoms and lifestyle advice, such as correct fluid intake, diet, and regular voiding time and bowel habits. Specific urotherapy includes specific treatment such as pelvic floor muscle training, pelvic floor biofeedback and electrical stimulation, alarm treatment, and neuromodulation [24] and is recommended in case of first line treatment failure. Moreover, urodynamic patterns may vary over time and sometimes tests can be normal in symptomatic patients (this situation is estimated to be present in 1–34% of patients) [20,25,26,27].

Currently, there are limited data about urological manifestation in MS pediatric patients in the literature. Scheepe et al. described the presence of LUTD in children affected by MS, especially in those with a higher Expanded Disability Status Scale (EDSS) score [10], correlating them with greater disease severity; the authors reported 5 out of 24 MS patients (21%) to have voiding disfunction [10]. In our series, five patients exhibited urinary incontinence and one showed retention symptoms; urodynamic exams revealed both OAB and underactive detrusor patterns.

In adult MS, pelvic floor muscle training (PFMT) and percutaneous tibial nerve stimulation (PTNS) are successfully used. A recent review showed that PFMT is a successful treatment option in women with MS for lowering the impact of urine incontinence and overactive bladder symptoms and increasing health-related quality of life. These results are greater if exercises are performed with the aid of a professional figure, for example a physiotherapist, and using BFB [28]. Wang et al. highlighted the importance of pelvic floor muscle electrotherapy to manage OAB. Electrical stimulation prevents involuntary contractions by inhibiting detrusor contractions [29]. In the literature, there are no studies about PFMT in pediatric MS. Only one of our patients performed PFMT with the aim of utilizing BFB and electrostimulation with poor results, maybe because of the small number of sessions. In adult patients with neurological symptoms, a 12-session course of therapy is recommended, even if there are no standardized protocols [30,31,32,33].

PTNS in adult patients with MS with LUTD and OAB is an effective treatment, particularly in non-responders to first line therapy [34,35]. This treatment is well tolerated in children affected by LUTD [36] but its use in children with MS has not been studied. In our experience, only one patient was scheduled for PTNS, but she refused this treatment.

However, we encountered initial resistance to urodynamic evaluation and/or starting CIC from patients and their parents; these events could therefore delay urological diagnosis and/or subsequent therapy, increasing the risk of future kidney damage. Indeed, the literature shows a prevalence of urinary tract complications of up to 40% in studies with 18-year follow-ups. These may include lower urinary tract infections (13–80%); bladder alterations, such as the formation of diverticula or trabeculae (4–49%); upper urinary tract infections (0–23%); hydronephrosis (0–25%); vesicoureteral reflux (0–15%); and stones (2–11%). The risk of renal failure (2–3%) does not appear to be greater in MS patients than in the general population. A greater risk of bladder cancer has been reported in adult MS patients compared to the general population, especially if subjected to immunosuppressants or catheterization (using intermittent or indwelling catheters) [20,37,38].

Currently, for patients who do not complain of urinary symptoms, a urological evaluation by a neurologist is considered sufficient, based on a careful anamnesis with questionnaires aimed at analyzing the presence of signs and/or symptoms of urological alteration and the evaluation of post-void residual. Particular attention should be paid during these visits as patients with MS often under-report urological symptoms. In case of urological anomaly detection, the neuro-urologist must conduct an in-depth evaluation, including, for example, bladder diaries, urinary tract ultrasound, the assessment of post-void residual, urinalysis, a blood exam to assess renal function, urodynamic tests (invasive and/or non-invasive exams), and cystoscopy [20].

We believe it is appropriate to initially perform a complete non-invasive assessment, with questionnaires, diaries, flowmetry, urinary ultrasounds, blood/urine exams, surface EMG, and post-void bladder residual evaluation (e.g., using a portable bladder scan in an outpatient setting) [39], and limit the execution of more invasive exams, such as invasive urodynamic examinations, cystourethrography, and cystoscopy, to patients who show altered urological conditions.

The psychological state of these patients must also be considered in the decision of when and how to investigate comorbidities [40,41]; this must be considered a worsening cause after MS diagnosis.

In our experience, urological manifestations occur simultaneously with the appearance of neurological symptoms or with a temporal latency of up to five years from diagnosis. Considering the significant variability in the onset of urological symptoms in MS and the fact that bladder dysfunction is considered a major cause of morbidity and hospitalization in these patients [11], we advocate that an early urological evaluation after the diagnosis of MS is useful. The use of urological questionnaires during neurological evaluation can be useful to detect urological problems in time, also leading to a greater awareness of patients and their parents about possible urological issues, allowing a timely intervention before urological problems worsen patients’ quality of life and become more difficult to manage.

In this way, it is possible to achieve an early diagnosis and promptly start urological management, achieving greater patient compliance and avoiding the deterioration of the urinary tract or the need for urgent invasive procedures.

Even colorectal dysfunction should not be underestimated, as digestive disorders are common in MS patients (45–68%). These symptoms, like urological ones, must be investigated carefully as patients often feel ashamed to report them or underestimate them. Intestinal problems can be divided into “retentive” (31–54%), with constipation and frequent abdominal pain, for example, or “irritative” (6–20%), including defecation urgency, diarrhea, and incontinence [42]. In our research, three out of six patients had obstinate constipation.

Therefore, a multidisciplinary team with a neurologist and neurorehabilitation doctor, pediatric urologist, pediatric digestive surgeon/gastroenterologist, psychologist, and urotherapist is highly beneficial.

## 5. Conclusions

As in the adult population, MS at a pediatric age is characterized by the presence of neurological and urological symptoms. The latter can manifest themselves with great variability, both in terms of type and timing of onset. We therefore believe that a urological assessment is important following any diagnosis of MS.

Although considered a rare diagnosis at a pediatric age, MS must be suspected in adolescents with LUTD associated with certain neurological manifestations.

The pediatric population diagnosed with MS is highly vulnerable to cognitive impairment, that may be a cause of depression or other psychological disturbances beyond physical disability and may contribute to patients’ low functional outcomes [20]. For these reasons, multidisciplinary management is suggested, involving a neurologist, physical rehabilitation specialist, physician, psychologist, pediatric urologist, and pediatric digestive surgeon/gastroenterologist, to define individualized rehabilitative treatment, improving MS complication management, quality of life, and psychosocial care.

This work was carried out within the European Reference Network for Rare Urogenital Diseases and Complex Conditions (ERN EUROGEN).

## Figures and Tables

**Table 1:** Criteria for diagnosis of pediatric MS according to Krupp criteria 2012 [12].

Pediatric MS (can be satisfied by any of the following)
Two or more non-encephalopathic (e.g., not ADEM-like) clinical CNS events with presumed inflammatory cause, separated by more than 30 days and involving more than one area of the CNS.
One non-encephalopathic episode typical of MS, associated with MR findings that are consistent with the 2010 Revised McDonald criteria for DIS, in which a follow-up MR shows at least one new enhancing or non-enhancing lesion consistent with the MS dissemination in time (DIT) criteria.
One ADEM attack followed by a non-encephalopathic clinical event, three or more months after symptom onset, which is associated with new MR lesions that fulfill the 2010 Revised McDonald DIS criteria.
A first, single, acute event that does not meet the ADEM criteria and whose MR findings are consistent with the 2010 Revised McDonald criteria for DIS and DIT (applies only to children ≥12 years old).

ADEM: acute disseminated encephalomyelitis; CNS: central nervus system; MR: magnetic resonance imaging; DIS: dissemination in space; DIT: dissemination in time.

**Table 2:** Definitions of dissemination in space and time for application of the 2010 McDonald Criteria [13].

Dissemination in Space	Dissemination in Time
≥1 T2 lesions in ≥2 of the following areas: Periventricular Juxtacortical Infratentorial Spinal cord	Simultaneous presence of gadolinium-enhancing and non-enhancing lesions on MR at any time or ≥1 new T2 and/or gadolinium-enhancing lesion on a follow-up MR, irrespective of its timing with reference to a baseline scan

MR: magnetic resonance imaging.

**Table 3:** Patient characteristics and follow-ups.

Pt No; Sex	Age at Diagnosis	LUTS Onset	LUTS (Type)	UD Exam	Urological Therapy	Urological FU	Bowel
1; female	13.1 yrs	Already present at MS diagnosis	Urge incontinence + enuresis	OAB, reduced BC high pressure	Standard urotherapy; CIC+ oxybutynin + BFB; BTX-A injections	2.4 yrs; improvement	Constipation; no therapy
2; female	15.6 yrs	16 yrs	Urinary retention	Dyssynergy and high PVR	Standard urotherapy; CIC	3.7 yrs; improvement	Constipation; laxatives + TAI
3; male	17.3 yrs	Already present at MS diagnosis	Incontinence	Acontractile bladder and low capacity	Standard urotherapy; CIC + oxybutynin; BTX-A	5.2 yrs; improvement	Constipation; laxatives + TAI
4; female	13.1 yrs	16.11 yrs	Incontinence	Not Invasive UD: interrupted flow + high PVR	Not performed	Lost at FU	None
5; female	16.8 yrs	17.6 yrs	Incontinence	Scheduled	Standard urotherapy	Recent referral to our unit	None
6; male	11.4 yrs	16.3 yrs	Urgency + Urge incontinence	Not Invasive UD: interrupted flow + high PVR	Standard urotherapy	2.4 yrs: resolution	None

Pt no: patient number; yrs: years; LUTS: lower urinary tract symptoms; UD: urodynamic; OAB: overactive bladder; BC: bladder capacity; BFB: biofeedback; PVR: post-void residual; FU: follow-up; TAI: trans-anal irrigation.

## Data Availability

The data presented in this study are available on request from the corresponding author due to ethical reasons.
